# Time to diagnosis in juvenile idiopathic arthritis: a french perspective

**DOI:** 10.1186/s13023-017-0586-4

**Published:** 2017-02-28

**Authors:** Laura Aoust, Linda Rossi-Semerano, Isabelle Koné-Paut, Perrine Dusser

**Affiliations:** 1 Division of Pediatric Rheumatology, Bicêtre Hospital, APHP, University of Paris SUD, 78 rue du Général Leclerc, Le Kremlin-Bicêtre, Cedex 94270 France; 2 Reference Center for Auto-inflammatory Disorders CEREMAI, Paris, France

**Keywords:** Juvenile arthritis, Health surveys, Arthritis, Juvenile/diagnosis*, Retrospective studies, Rheumatology, Referral and consultation/statistics & numerical data*, Pediatrics

## Abstract

**Background:**

Juvenile idiopathic arthritis (JIA) is a rare disease that is not widely known by paediatricians and general practitioner (GP) leading to diagnostic error and delayed care provision. We aimed to analyse patient’s journey and time to diagnosis of JIA (delay from the first symptom to the diagnosis of JIA).

We performed a retrospective cohort study of 67 patients diagnosed with JIA and seen in the paediatric rheumatology department of the Kremlin Bicêtre Hospital, between July 2002 and January 2015. Patients were selected for analysis in order to represent an equal distribution of five JIA subtypes: oligoarticular onset (21), polyarticular onset (13), enthesitis-related arthritis (17), and systemic onset (16).

**Results:**

Sixty-seven patients were finally analysed (42 girls). Before JIA diagnosis was made, patients had visited a mean of three physicians (3.6 ± 1.4 (mean; SD)). Emergency room physicians (52%) were the first patient’s referral before GP (42%). Paediatric rheumatologists were mostly seen as third referral (52% versus 3% at first referral). Reactive arthritis (34%) and septic arthritis (24%) represented both the most common initial diagnosis. JIA was suspected after an average median time delay of 3 months (0.26–81.2) except for 25 patients (37%): SJIA (*n* = 9), ERA (*n* = 7), OAJIA (3) and POJIA (*n* = 6) for whom diagnosis was suspected straightaway. In most cases (88%), JIA was established by paediatric rheumatologists.

Surprisingly, the median total time to diagnosis in our population was rather short (3 months). Paediatric rheumatologist played a major role in making the diagnosis but the journey to reach them was long and complex with multiple referrals. These results reinforce the necessity of improving GP and emergency physician’s awareness and education on paediatric rheumatic diseases as the importance of a strong network in paediatric rheumatology to improve patient’s level of care.

**Conclusion:**

We highlighted the complex patient’s journey to diagnosis in children with JIA and made assumptions that reference center might reduce time to diagnosis although not statically proven. Further analysis with a larger number of patients might be needed to better investigate this probability.

## Background

Juvenile idiopathic arthritis, JIA, is the most frequent cause of inflammatory arthritis in children before 16 years. Even if considered a rare disease, it’s estimated incidence rate is different around the world with low incidence in Asian population and relatively higher incidence in those of European descent (0.83 per 100,000 children in Japan to 23 per 100,000 in Norway per year) [1, 2]. Only few surveys have assessed epidemiology of JIA in France but nothing is known on patient’s journey to accurate diagnosis and care. JIA is currently classified into seven subtypes according to the Edmonton consensus meeting 2001 [3]. Even if paediatric rheumatology is still considered a young subspecialty in many countries, the standards of medical education and patient’s care have considerably evolved with time [4]. In contrast to that, the academic recognition of this subspecialty and the opportunities to get appropriate medical education remain clearly insufficient in many European countries. Indeed, until the early years 2000, the whole paediatric rheumatology expertise represented a maximum of five centres in France. At the same time, in other European countries and in the United states, the evolution of concepts and the use of biological treatments had considerably modified the patient’s care toward highly specialized multidisciplinary paediatric rheumatology teams [5, 6]. In 2004, the French health minister initiated a national campaign for rare diseases that has dramatically changed the recognition of paediatric rheumatology. Indeed, this campaign lead to the labelling of two reference centres in 2006 and 2007 with a network of 18 competence centres throughout the national territory [7]. We can hypothesise that this new organization has increased the quality of care for patients with rheumatic diseases, with a growing number of opportunities for young paediatricians to access accurate medical education in paediatric rheumatology. Despite this new organisation, paediatricians and general practitioner (GP) have insufficient knowledge regarding rheumatic diseases and paediatric musculoskeletal clinical examination [8] probably leading to an underestimation of the actual number of JIA patients in France. As joint symptoms are the most common initial complaints at disease onset, we hypothesized that the lack of knowledge underlies diagnostic error - a great source of suffering for patients and their families - and delayed care provision. To better assess the remaining unmet needs in our country, we aimed to analyse patient’s journey and time to diagnosis of JIA.

## Methods

We performed a retrospective cohort study of patients diagnosed with JIA according to the ILAR criteria and seen in the paediatric rheumatology center of Kremlin Bicêtre, a single tertiary and reference center for paediatric rheumatology in France, between July 2002 and January 2015. Patients were randomly selected for analysis and classified in five JIA subtypes: e.g. oligoarticular onset (OAJIA), oligo-onset (persistent and extended) polyarticular onset (POJIA) (rheumatoid factor (RF) positive and negative), enthesitis-related arthritis (ERA), and systemic onset (SoJIA). In addition, sufficient information on patient’s journey to diagnosis and care was required.

We collected demographic clinical and biological information (C-reactive protein level, erythrocyte sedimentation rate, antinuclear antibodies (ANA), RF and HLA-B27) where available. We recorded also all major medical investigations and referrals, treatments and differential diagnoses made before diagnosis confirmation. The time to diagnosis was defined as the delay from the first symptom to the diagnosis of JIA. According to our national regulations, no IRB approval was required.

The cohort’s characteristics, time to diagnosis, and other variables were analysed using descriptive statistics. Total time to diagnosis was secondarily adjusted on sex, age and number of symptoms at disease onset, type of JIA, first referral physician consulted and the distance between home and reference center using a multivariate logistic regression.

## Results

### All patients

Sixty-seven patients were finally analysed (25 boys; M/F SR: 0.6): 21 were diagnosed with OAJIA, 16 with SoJIA, 17 with ERA and 13 with POJIA. Mean age at disease onset was of 6.4 ± 4.6 years (mean; SD) (0.5–15.6). The whole population characteristics are summarized in Table 1.

**Table 1:** Population characteristics

	Sex (F/M)	Age at disease onset (years; mean ± SD)	Min-Max (years)	ANA+	RF+	HLAB27
Oligoarticular JIA	15/6	3,6 ± 2,9	1–11,6	38%	0%	12%
Polyarticular JIA	12/1	5,7 ± 4,2	0,9–13	54%	10%	0%
ERA JIA	8/9	10,3 ± 4,2	1,5–15,6	18%	0%	44%
Sytemic JIA	7/9	6,4 ± 4,3	0,4–15	25%	0%	0%
Total	42/25	6,4 ± 4,6	0,5–15,6	42%	10%	33%

*JIA* juvenile idiopathic arthritis, *F* female, *M* male, *JIA* juvenile idiopathic arthritis, *ANA* Antinuclear antibody, *RF* Rheumatoid factor

**Fig. 1:**
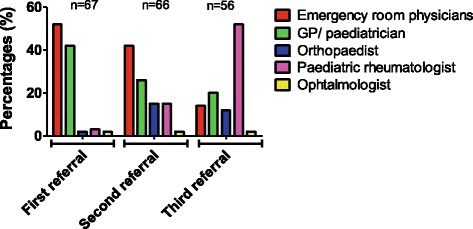
Graph representing the different physicians seen at first, second and third referral

**Fig. 2:**
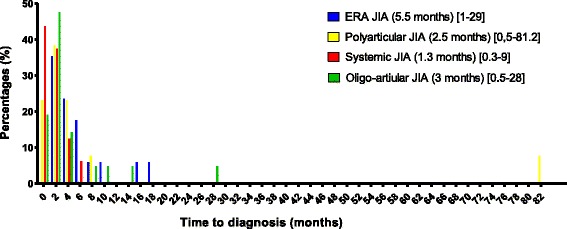
Histogram showing time to diagnosis (months) according to each juvenile idiopathic arthritis (JIA) subgroups. Median time to diagnosis are given for each JIA subtypes (months)

The most common symptoms that led to seek medical attention at disease onset, were arthralgia (39%), arthritis (25%), and fever (18%) followed by skin rash (8%), limping (9%), abdominal pain (1.3%), and uveitis (1.3%). Before JIA diagnosis was made, patients had visited a mean of three physicians (3.6 ± 1.4 (mean; SD)). Emergency room physicians (52%) were the first patient’s referral before the general practitioner (42%) (Fig. 1). Reactive arthritis (34%) and septic arthritis (24%) represented both the most common initial diagnosis. Fifty-two patients (78%) underwent radiological exams, in which X-rays (49%) and articular ultrasounds (36%). Twelve patients (17%) had joint fluid aspiration. Nineteen patients (28%) received antibiotics; mostly amoxicillin plus clavulanic acid (13.4%). JIA was suspected after an average median time delay of 3 months (0.26–81.2) except for 25 patients (37%): SJIA (*n* = 9), ERA (*n* = 7), OAJIA (3) and POJIA (*n* = 6) for whom diagnosis was suspected straightaway (Fig. 2). In most cases (88%), JIA was established by paediatric rheumatologists.

### Systemic onset JIA

**Fig. 3:**
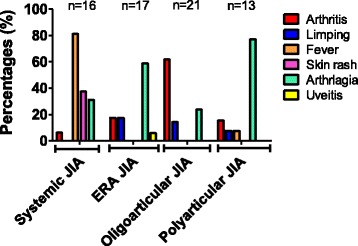
Graph showing the presenting symptoms at disease onset for each juvenile idiopathic arthritis (JIA) subtypes

Sixteen patients had SoJIA (9 boys; M/F SR: 1.28). Age at disease onset was of 6.4 ± 2.5 years (mean; SD) (0.4–15.2) and initial symptoms included fever 81%, skin rash 37.5%, and arthralgia 31% (Fig. 3). At first referral, the most frequently consulted physicians were GP’s (56%) and emergency department physicians (37%) with a mean of 1.6 hospitalizations. SoJIA was diagnosed out of hand in 43.7% of cases. Different diagnoses were discussed before JIA was diagnosed such as: reactive arthritis (31%), septic arthritis (12.5%) and osteomyelitis (12.5%). X-rays were performed in half cases, joint ultrasounds in 31% of cases and bone scintigraphy in 25% of cases. Fifty-six per cent of patients received antibiotics. Paediatric rheumatologists made the diagnosis of SoJIA in 87.5% of cases and within a median period of 1.3 months (0.3–9).

### Oligoarthritis onset JIA

Twenty-one patients (6 boys; M/F SR: 0.4) started their disease at 3.6 ± 2.9 years (mean; SD) (1.08-11.6). Arthritis (62%) and arthralgia (24%) were the main presenting symptoms (Fig. 3). Emergency physicians (71%) were the most consulted at first referral and patients had a mean of 0.7 hospitalizations. Septic arthritis (52%) and reactive arthritis (43%) were initially diagnosed although no patients presented with fever. JIA was initially suspected in three patients (14%). X-rays were performed in 67% and joint ultrasound in 57%. Patients had received antibiotics in 38% of cases even in patients without evidence of bacterial infection at joint aspiration fluid analysis (38%). OAJIA patients were the most investigated due to monoarticular presentation (57% of monoarthritis) at disease onset. Orthopaedics cares (25%) were therefore provided leading to hospitalisation (67%), arthrocentesis (38%) performed, in most cases, under general anaesthetic due to age, arthrotomy (5%) and arthroscopic synovectomy (5%). JIA diagnosis was made by paediatric rheumatologist in 76% of cases with a median delay of 3 months (0.5–28).

### Polyarthritis onset JIA

Thirteen patients (5 boys; M/F SR: 0.6), started their disease at 5.7 ± 4.2 years (mean; SD) (0.9–13). Arthralgia (77%) and arthritis (15.4%) were the main presenting symptoms (Fig. 3). JIA was suspected at first referral to a physician in most cases (46%). GP and emergency physicians were the first consulted physicians with a mean of 0.7 hospitalisations. X-rays were performed in 54% of cases and joint ultrasound in 15% of cases. In some cases, (31%), patients had received prior antibiotics. Paediatric rheumatologist made the diagnosis in 92% of cases, within a median delay of 2.5 months (0.5–81.2).

### ERA onset JIA

Seventeen patients (9 boys; M/F SR: 1.1) started their disease at 10.4 ± 4.2 years (mean; SD) (1.5–15.6). Initial symptoms were mostly arthralgia (59%), arthritis (17.6%) and limping (17,6%) (Fig. 3). Septic and reactive arthritis were the two most frequent initially suspected diagnoses. Among reactive arthritis, 23.5% of patients were first diagnosed with transient hip synovitis. As in other JIA subgroups, emergency physicians (53%) and GP (35%) were the first referrals. JIA ERA patients had more radiological exams (82%), such as MRI (41%) and joint ultrasound (29%). Unlike in other JIA subtype, JIA ERA patients were exclusively diagnosed by paediatric rheumatologist within a median delay of 5.5 months (1–29) after a mean of 0,6 hospitalisations.

## Discussion

This study analyses a representative cohort of JIA patients affected by four main JIA subtypes [9]. Surprisingly, the median total time to diagnosis in our population was rather short (3 months) considering that JIA is characterized by arthritis persisting for more than six weeks. No factors influencing diagnosis delay were shown in multivariate analysis probably due to a too small population. Children with systemic-onset JIA had the shortest delay to diagnosis. Indeed, i.e. prolonged fever associated to intense constitutional symptoms, generally lead to hospitalization and accelerated paediatric rheumatology referral. As expected, total time to diagnosis of ERA was the longest and paediatric rheumatologists made the diagnosis in all cases. Indeed, making a relationship between back pain and possible inflammatory arthritis as well as recognizing enthesitis requires appropriate specialized referral. In Weiss and al. study, 66% of JIA patients had tender entheses with a median number of 2 tender entheses at initial evaluation. Enthesitis were more often symmetrical and affected mostly the patellar ligament insertion, the plantar fascial insertion at the calcaneus, the Achilles tendon insertion at the calcaneus and the plantar fascial insertion at the metatarsal heads [10]. In addition, GP or general paediatrician almost always consider inflammatory hip pain in children as transient synovitis, even though this diagnosis should remain of exclusion and limited to younger patients (mean 5.6 years old; rage: 1–13) [11]. In Rostom’s study, hip arthritis was described in 40 out of 121 JIA patients (33%) especially in ERA and polyarticular JIA. Hip pain appeared at an average age of 24 ± 10 years (3–46 years) in Rostom and al.’s study [12] and at a mean age of 10 ± 4 years in our JIA cohort.

Paediatric rheumatologist played a major role in making the diagnosis but the journey to reach them was long (6 months on average) and complex with multiple referrals (99%), unjustified diagnostic procedures and inappropriate diagnoses [13, 14]. Similar observations were reported by Feder and al. [13]. In the absence of trauma or sepsis, JIA is the most likely diagnosis in a child presenting with a monoarthritis, and arthroscopy and synovial biopsy are rarely warranted [13]. Imaging such as computerized tomography and isotope bone scans incur considerable radiation exposure; magnetic resonance imaging and ultrasound are often used but the diagnosis of JIA can usually be made without these tests. In addition to multiple investigations, patient received multiple courses of medication namely antibiotics. Diagnostic criteria for septic arthritis (SA) should be strictly abided by clinical and biological features such as: weight-bearing status, CRP >20 mg/L [15] and joint aspiration bacteriological results, to minimize needless antibiotic. In our study, all patients with joint aspiration were treated with antibiotics for a period of 4 to 6 weeks although no sign of infection were found. This attitude is unfortunate knowing how the misuse of antibiotic can be damaging. Some studies suggest that environmental factors impacting the composition of the microbiota, such as delivery mode and early exposure to antibiotics, affect the risk of chronic inflammatory diseases including JIA [16, 17]. For all these reasons, our study reinforces the necessity of improving GP and emergency physician’s awareness and education on paediatric rheumatic diseases [8, 18]. In addition, the complexity of JIA patient’s journey to accurate diagnosis and care allows us to highlight the importance of a strong network in paediatric rheumatology to improve patient’s level of care. At the European level, strong networks have been created in the past few years in order to support recommendations of care, research and patient’s education of paediatric rheumatology: Paediatric Rheumatology European Society (PRES), Paediatric Rheumatology International Trials Organisation (PRINTO) together with the EULAR Standing Committee on Paediatric Rheumatology [4, 5].

Our study represents a first attempt to determine the level of wandering in JIA diagnosis in a country which has been the first to increase the visibility of care for rare diseases by creating reference centres. Although the distance between home and the reference center was not found to correlate with time to diagnosis, we made assumptions that it could be one reason for rather earlier referral than previously reported in other Western countries; indeed, three-fourths of patients referred to us for JIA are living in Paris and the “Ile de France” region. An analysis with larger number of patients might be needed to better investigate this probability. Nevertheless, other factors could play a key role in reducing time to diagnosis such as elevated ESR, type of JIA and presence of enthesitis. Larger studies are needed to evaluate these parameters. However, the apparent limited median delay to diagnosis observed herein must be dampened by the wide ranges, up to 86 months in POJIA. In addition, part of the collection of data was retrospective including somewhat recall biases.

## Conclusion

In conclusion, we have highlighted the complex patient’s journey to diagnosis in JIA, possibly improved by our reference center effect although no significant correlation was found. The limited number of patients presented calls for a nationwide survey.

## Abbreviations

ANA: Antinuclear antibodies
ERA: Enthesitis-related arthritis
GP: General practitioner
JIA: Juvenile idiopathic arthritis
OAJIA: Oligoarticular onset Juvenile idiopathic arthritis
POJIA: Polyarticular onset Juvenile idiopathic arthritis
PRES: Paediatric Rheumatology European Society
PRINTO: Paediatric Rheumatology International Trials Organisation
RF: Rheumatoid factor
SoJIA: Systemic onset Juvenile idiopathic arthritis

## References

[1] Berntson L, Andersson Gäre B, Fasth A, Herlin T, Kristinsson J, Lahdenne P (2003). Incidence of juvenile idiopathic arthritis in the Nordic countries. A population based study with special reference to the validity of the ILAR and EULAR criteria. J Rheumatol.

[2] Thierry S, Fautrel B, Lemelle I, Guillemin F (2014). Prevalence and incidence of juvenile idiopathic arthritis: a systematic review. Joint Bone Spine.

[3] Petty RE, Southwood TR, Manners P, Baum J, Glass DN, Goldenberg J (2004). International League of Associations for Rheumatology classification of juvenile idiopathic arthritis: second revision, Edmonton, 2001. J Rheumatol.

[4] Gäre BA (2001). EULAR standing committee on paediatric rheumatology. Ann Rheum Dis.

[5] Ruperto N, Martini A (2011). Networking in paediatrics: the example of the Paediatric Rheumatology International Trials Organisation (PRINTO). Arch Dis Child.

[6] Spencer CH (2007). Why should pediatric rheumatology be recognized as a separate subspecialty: an open letter to medical councils and government agencies. Pediatr Rheumatol Online J.

[7] Les Cahiers d'Orphanet - Maladies Rares Info. Serviceswww.maladiesraresinfo.org/…/pdf/Liste_des_centres_de_reference_labellises_maladie…2. oct. 2010- http://www.orpha.net/orphacom/cahiers/docs/FR/Liste_des_centres_de_reference_labellises_maladies.pdf. série Politique de santé. 2.

[8] Myers A, McDonagh JE, Gupta K, Hull R, Barker D, Kay LJ (2004). More “cries from the joints”: assessment of the musculoskeletal system is poorly documented in routine paediatric clerking. Rheumatology (Oxford).

[9] Gowa MA, Ibrahim MN, Memon BN, Raza SJ (2015). A cross sectional study on juvenile idiopathic arthritis in paediatric population. J Pak Med Assoc.

[10] Weiss PF, Klink AJ, Behrens EM, Sherry DD, Finkel TH, Feudtner C (2011). Enthesitis in an inception cohort of enthesitis-related arthritis. Arthritis Care Res.

[11] Dubois-Ferrière V, Belaieff W, Lascombes P, de Coulon G, Ceroni D (2015). Transient synovitis of the hip: which investigations are truly useful?. Swiss Med Wkly.

[12] Rostom S, Amine B, Bensabbah R, Abouqal R, Hajjaj-Hassouni N (2008). Hip involvement in juvenile idiopathic arthritis. Clin Rheumatol.

[13] Foster HE, Eltringham MS, Kay LJ, Friswell M, Abinun M, Myers A (2007). Delay in access to appropriate care for children presenting with musculoskeletal symptoms and ultimately diagnosed with juvenile idiopathic arthritis. Arthritis Rheum.

[14] Tzaribachev N, Benseler SM, Tyrrell PN, Meyer A, Kuemmerle-Deschner JB (2009). Predictors of delayed referral to a pediatric rheumatology center. Arthritis Rheum.

[15] Singhal R, Perry DC, Khan FN, Cohen D, Stevenson HL, James LA (2011). The use of CRP within a clinical prediction algorithm for the differentiation of septic arthritis and transient synovitis in children. J Bone Joint Surg Br.

[16] Horton DB, Scott FI, Haynes K, Putt ME, Rose CD, Lewis JD (2015). Antibiotic exposure and juvenile idiopathic arthritis: a case–control study. Pediatrics.

[17] Arvonen M, Berntson L, Pokka T, Karttunen TJ, Vähäsalo P, Stoll ML (2016). Gut microbiota-host interactions and juvenile idiopathic arthritis. Pediatr Rheumatol Online J.

[18] Sen ES, Clarke SLN, Ramanan AV (2014). The child with joint pain in primary care. Best Pract Res Clin Rheumatol.

